# Secreting Germ Cell Tumors of the Central Nervous System: A Long-Term Follow-up Experience

**DOI:** 10.3390/cancers12092688

**Published:** 2020-09-21

**Authors:** Veronica Biassoni, Elisabetta Schiavello, Lorenza Gandola, Emilia Pecori, Geraldina Poggi, Filippo Spreafico, Monica Terenziani, Cristina Meazza, Marta Podda, Andrea Ferrari, Roberto Luksch, Michela Casanova, Nadia Puma, Stefano Chiaravalli, Luca Bergamaschi, Graziella Cefalo, Fabio Simonetti, Giovanna Gattuso, Ettore Cesare Seregni, Federica Pallotti, Francesca Gianno, Barbara Diletto, Francesco Barretta, Maura Massimino

**Affiliations:** 1 Pediatric Oncology Unit, Fondazione IRCCS Istituto Nazionale dei Tumori, 20133 Milan, Italy; elisabetta.schiavello@istitutotumori.mi.it (E.S.); filippo.spreafico@istitutotumori.mi.it (F.S.); monica.terenziani@istitutotumori.mi.it (M.T.); cristina.meazza@istitutotumori.mi.it (C.M.); marta.podda@istitutotumori.mi.it (M.P.); andrea.ferrari@istitutotumori.mi.it (A.F.); roberto.luksch@istitutotumori.mi.it (R.L.); michela.casanova@istitutotumori.mi.it (M.C.); nadia.puma@istitutotumori.mi.it (N.P.); stefano.chiaravalli@istitutotumori.mi.it (S.C.); luca.bergamaschi@istitutotumori.mi.it (L.B.); fabio.simonetti@istitutotumori.mi.it (F.S.); giovanna.gattuso@istitutotumori.mi.it (G.G.); maura.massimino@istitutotumori.mi.it (M.M.); 2 Pediatric Radiotherapy Unit, Fondazione IRCCS Istituto Nazionale dei Tumori, 20133 Milan, Italy; lorenza.gandola@istitutotumori.mi.it (L.G.); emilia.pecori@istitutotumori.mi.it (E.P.); barbara.diletto@istitutotumori.mi.it (B.D.); 3 Neuro-Oncological Unit and Neuropsychological Rehabilitation Unit Scientific Institute, IRCCS E. Medea, Bosisio Parini, 23842 Lecco, Italy; geraldina.poggi@lanostrafamiglia.it; 4 Department of Pediatrics, University of Milan, San Paolo Hospital, Santi Paolo e Carlo ASST, 20121 Milan, Italy; graziella.cefalo@asst-santipaolocarlo.it; 5 Nuclear Medicine Unit, Fondazione IRCCS Istituto Nazionale dei Tumori, 20133 Milan, Italy; ettore.seregni@istitutotumori.mi.it (E.C.S.); federica.pallotti@istitutotumori.mi.it (F.P.); 6 Department of Radiological, Oncological and Anatomo-Pathological Sciences, University Sapienza of Rome, 00161 Rome, Italy; francesca.gianno@uniroma1.it; 7 Clinical Epidemiology and Trial Organization Unit, Fondazione IRCCS Istituto Nazionale dei Tumori, 20133 Milan, Italy; francesco.barretta@istitutotumori.mi.it

**Keywords:** CNS germ cell tumors, NGGCT, chemotherapy, radiotherapy, craniospinal irradiation, late effects

## Abstract

**Simple Summary:**

Nongerminomatous germ cell tumors of the central nervous system are rare tumours. Differently from germinomas, they have a severe prognosis above all when presenting with high alfafetoprotein levels. We report the results of a combined chemo- and radiotherapy approach in 28 patients affected by this disease with craniospinal irradiation and a boost tailored on the response to pre-radiant chemotherapy. Metastatic patients and high-risk disease are discussed as well. The 5 years overall survival and event-free survival were both 81% while at 10 years they were 81% and 76% respectively. Our series, even if small, concerns nongerminomatous germ cell tumors only (whereas in some papers they are mixed with pure germinomas), furthermore our patients had a very long follow-up (over 11 years) with encouraging survival data for localized and metastatic disease. Improving survival while trying to contain/avoid the long-term sequelae of chemotherapy and radiotherapy are the main goals of future studies.

**Abstract:**

Introduction: Due to the rarity of nongerminomatous germ cell tumors (NGGCT) with non-standard treatment as yet, we report retrospectively our 30 year experience with chemotherapy followed by craniospinal irradiation (CSI), plus a boost of whole ventricular irradiation (WVI)/tumor bed (TB), tailored to pre-radiation chemotherapy response. Methods: Between 1988 and 2016, 28 patients received four cycles of PEB (cisplatin/etoposide/bleomycin), then CSI, and two further PEB cycles. Between 1988 and1994, CSI was 25.5 Gy for patients in complete remission (CR), 30 Gy if in partial remission (PR) or metastatic, with a boost to TB up to 45–54 Gy. In the period of 1995–2010, the boost included WVI and any extra-ventricular tumor sites up to 45 Gy. After 2010, CSI was reduced to 25.5 Gy for all non-metastatic patients, and a boost was given only to TB up to 40.5/45.5 Gy, depending on patients’ CR/PR status. After 2003, patients with alfafetoprotein (αFP) > 1000 ng/mL received intensified treatment, also including autologous stem cell transplantation. Results: Among 28 patients (23 males; median age 12 years, 6 metastatic), 25 responded to PEB, and three progressed (PD) after one to four cycles; 26 received radiotherapy obtaining 13 CR, 7 PR and 5 stable disease (SD), 1 PD; 6 (21%) died (5 for disease, 1 for pneumonia while in CR). Five-year overall survival (OS) and progression-free survival (PFS) were both 81%; 10 year OS and PFS 81% and 76%, respectively (median follow-up 11 years). Conclusions: Survival for children with NGGCT, independently from disease extent, was encouraging. Further studies should elucidate which patients could benefit from reduced volume and dose irradiation.

## 1. Introduction

Intracranial germ cell tumors (GCTs) account for 0.3–0.5% of all central nervous system (CNS) tumors in Europe, while in Asia their incidence reaches 2–5% [1]. They typically occur in the midline, suprasellar or pineal regions of the brain, mainly in adolescence. GCTs in the basal ganglia, brainstem and spine have also been reported. They are traditionally divided into pure germinomas and nongerminomatous germ cell tumors (NGGCTs), which include embryonal carcinoma, yolk sac tumors (endodermal sinus tumor), choriocarcinoma, and mature/immature teratoma, often in various combinations. Between 1975 and 2000, the SEER 5 year survival probability for all GCTs was 81% for 8–14-year-olds, and 94% for patients aged 15–29 [2]. GCTs reportedly have a survival probability exceeding 90%, while NGGCTs carry a worse prognosis, the 5 year overall survival (OS) probability being 60–70% [3,4].

The 2016 WHO classification of CNS tumors made no substantial changes to this classification [5]. In the latest International Society of Paediatric Oncology (SIOP) CNS GCT II protocol, the established cutoff for considering a GCT as “secreting” (i.e., NGGCT), with a view to avoiding diagnostic surgery, was serum and/or cerebrospinal fluid (CSF) αFP > 25 ng/mL, or serum and/or CSF β-HCG > 50 IU/l [6].

The heterogeneity of NGGCTs is responsible for lower radiosensitivity than pure germinomas, with a 5 year OS probability of 20–45% with radiotherapy (RT) alone [7]. Most recent studies consequently combined platinum-based chemotherapy with RT, the fields, extent, total dose and fractionation of which have yet to be standardized [8,9,10,11,12]. PEB (cisplatin, etoposide, bleomycin) chemotherapy was introduced in the early 1980s [13]. Its use in CNS NGGCTs was explored in a German trial in 1986 [14], and in a subsequent European trial started in 1993 [15]. In these two experiences, CSI (30 Gy) plus a boost (24 Gy) to the TB reportedly achieved a 5 year event-free survival (EFS) of 67% and 81%, respectively. We used PEB as well as pre-radiant chemotherapy, and given the rarity of NGGCT, the paucity of published studies on it (most reported series included both germinomas and NGGCTs), and the still unsatisfactory prognosis, it seems useful to add our single-institution experience of a consecutive series of patients with NGGCT spanning nearly 30 years.

All procedures performed in this study were in accordance with the ethical standards of the institutional and/or national research committee and with the 1964 Helsinki declaration and its later amendments or comparable ethical standards. The institutional review boards reviewed and approved the protocol as 128/19.

## 2. Materials and Methods

Between 1988 and 2016, all consecutive patients with NGGCT were treated at our institution with a combined chemo-radiotherapy regimen. Patients were diagnosed according to their serum and cerebrospinal (CSF) αFP and β-HCG values, as defined by the SIOP with serum and/or CSF αFP > 25 ng/mL, or serum and/or CSF β-HCG > 50 IU/l [16].

### 2.1. Diagnostic Workup and Staging

All patients underwent MRI at diagnosis, with CSF cytology and CSF marker assessment via lumbar puncture unless it was contraindicated. Spinal MRI was always performed prior to any lumbar puncture. Localized disease was defined as a lesion with no evidence of cranial or spinal dissemination on MRI or CSF cytology. Bifocal disease with tumor locations in both the pineal and the suprasellar regions was not considered as metastatic. Metastatic disease was instead defined as >1 intracranial localization (other than bifocal disease), or spinal metastases, or metastases outside the CNS, or tumor cells in the CSF.

MRI and serum/CSF markers were reassessed after the 2nd chemotherapy cycle, after the 4th (before RT), after RT, and at the end of the treatment. When tissue samples were available, histopathological review was performed, at our institution until the year 2000, and by a national pathology referral center from then on.

### 2.2. Treatment

Treatment changed due to the long time span in the light of incoming literature data regarding different risk clinical features.

#### 2.2.1. Chemotherapy

In the period of 1988–2014, patients received 4 PEB cycles, then RT and 2 further PEB cycles. PEB consisted of cisplatin 25 mg/sqm and etoposide 125 mg/sqm on days 1 to 4, plus bleomycin 18 mg/sqm (maximum dose 30 mg) on day 2 of each cycle. The interval between cycles was 21 days before RT, and 21–28 days afterwards. After 2014, all patients received only the 4 courses of PEB before RT.

#### 2.2.2. Radiotherapy

In the period of 1988–1994, RT consisted of craniospinal irradiation (CSI), modulating the total dose on the response obtained after induction chemotherapy (25.5 Gy for biological and/or radiological complete remission [CR], 30 Gy for partial response [PR] or metastases at diagnosis) plus a boost to the tumor bed (TB) up to 45–54 Gy.

Between 1995 and 2010, the boost included whole ventricular irradiation (WVI) up to a total dose of 45 Gy, and also any extra-ventricular tumor sites revealed at diagnosis.

After 2010, the CSI dose was reduced to 25.5 Gy for all non-metastatic patients, with a boost only to the TB up to a total dose of 40.5 (for CR after chemotherapy) or 45.5 Gy (for PR). The fractionation adopted was always the same, i.e., 1.5 Gy/d for CSI, and 1.8 Gy/d for boosts.

### 2.3. High-Risk Patients

After 2003, chemotherapy was intensified for patients with αFP > 1000 ng/mL considered as having high-risk disease. This consisted of 2 PEB cycles followed by other sequential high-dose drugs, RT, and autologous stem cell transplantation previously harvested.

### 2.4. Follow-up

After completing the treatment, the routine clinical-oncological follow-up included a thorough clinical examination (neurological and functional assessment, vision, hearing, spirometry, and endocrine assessment), markers and neuroimaging. Spinal MRI was performed at every follow-up in cases of metastatic disease, or when suspicious symptoms developed, or whether serum markers increased. During the follow-up, markers were only tested in serum unless clinical signs suggested otherwise. Follow-ups were scheduled every 4 months for the first 2 years, then 6-monthly up to 5 years, and annually thereafter.

### 2.5. Neurocognitive Assessment

When feasible, data were also collected on neurocognitive functioning. The long accrual period meant that these data were not homogeneously available for all patients, however.

#### 2.5.1. Intellectual Abilities

The Third Edition of the Wechsler Intelligence Scale for Children (WISC-III) was used to assess patients’ full-scale (FSIQ), performance (PIQ), and verbal (VIQ) IQ. All these measures are expressed as standard scores.

#### 2.5.2. Attention

Sustained attention was examined with the Continuous Performance Test II (CPT-II), considering the overall index: scores below 8 on the CPT overall index indicate the absence of attention problems.

#### 2.5.3. Executive Functioning

Executive functioning was assessed with the Wisconsin Card Sorting Test (WCST), collecting adjusted standard scores for number of errors and number of perseverations.

#### 2.5.4. Memory

Long-term visuospatial memory was assessed with the Rey–Osterrieth Complex Figure Test for Memory (Rey-Memory), with performance expressed as z-scores.

#### 2.5.5. Visual-Motor Integration

Visual-motor integration was assessed with the Rey–Osterrieth Complex Figure Test for Copy (Rey Copy), with measures expressed as z-scores.

### 2.6. Statistical Analysis

Overall survival (OS) was defined as the time elapsing from diagnosis to death due to any cause. Time was censored as at the last follow-up for patients still alive. Progression-free survival (PFS) was defined as the time elapsing from diagnosis to disease progression/relapse or death, whichever came first. Time was censored at the latest follow-up for patients who were still alive and disease-free. OS and PFS curves were estimated using the Kaplan–Meier method, and compared using the log-rank test. The variables primary tumor site (localized—including bifocal—and metastatic), αFP levels (<1000 ng/mL, >1000 ng/mL), and metastatic status within 5 and 10 years were included in the analysis. Results were considered statistically significant for *p* values below 0.05 (*p* < 0.05).

## 3. Results

### 3.1. Diagnosis

Among the 28 patients, 23 were males and 5 were females; the median age at diagnosis was 12 years (range: 5–35 years). The mean duration of their symptoms before diagnosis was 275 days (range: 1 day–6 years): 10 patients were diagnosed within 1 month of the onset of symptoms, 15 after 1–12 months, and 3 after 36 months. Signs and symptoms prompting diagnostic imaging included: endocrine alterations (19 patients), increased intracranial pressure (6 patients), multiple cranial nerve palsy (2 patients with diplopia or campimetric deficits), sacral-coccygeal pain and lower limb weakness in 1 patient with spinal tumor. The median duration of symptoms for patients with signs of increased intracranial pressure was 60 days, while for those with endocrine dysfunctions, it was 330 days.

Patients’ characteristics are shown in Table 1. Seventeen out of 28 had localized disease (8 pineal, 8 supra-intrasellar, 1 spinal), 5 had bifocal, and 6 had metastatic disease (4 positive CSF cytology, 1 ventricular dissemination, 1 lung metastases).

Twenty patients underwent surgery at diagnosis: a CSF shunt in six patients, biopsy/surgical resection in eight, and both in six. The 12 patients requiring a CSF shunts had a ventricular-peritoneal device in 9 cases and a ventriculo-cysternostomy in 3. Tumor surgery consisted of total removal in four cases, subtotal in four, and biopsy alone in six patients.

Histology revealed all NGGCT except for two germinomas (but the specimens were very small in both, and markers were consistent with NGGCT). Fourteen out of 28 patients had a “biohumoral” diagnosis, based on markers and imaging alone. All but one patient (in poor clinical conditions due to uncontrolled diabetes insipidus and with pathological serum markers) underwent both CSF and serum markers assessment before starting chemotherapy.

Markers were distributed as shown in Table 2: 15/28 patients had serum αFP values > 25 ng/mL; 12 had CSF αFP > 25 ng/mL; 12 had serum β-HCG > 50 IU/L; and 18 had CSF β-HCG > 50 IU/L. Eight of the 28 patients had serum and/or CSF markers above 1000.

### 3.2. Treatment

**Figure 1:**
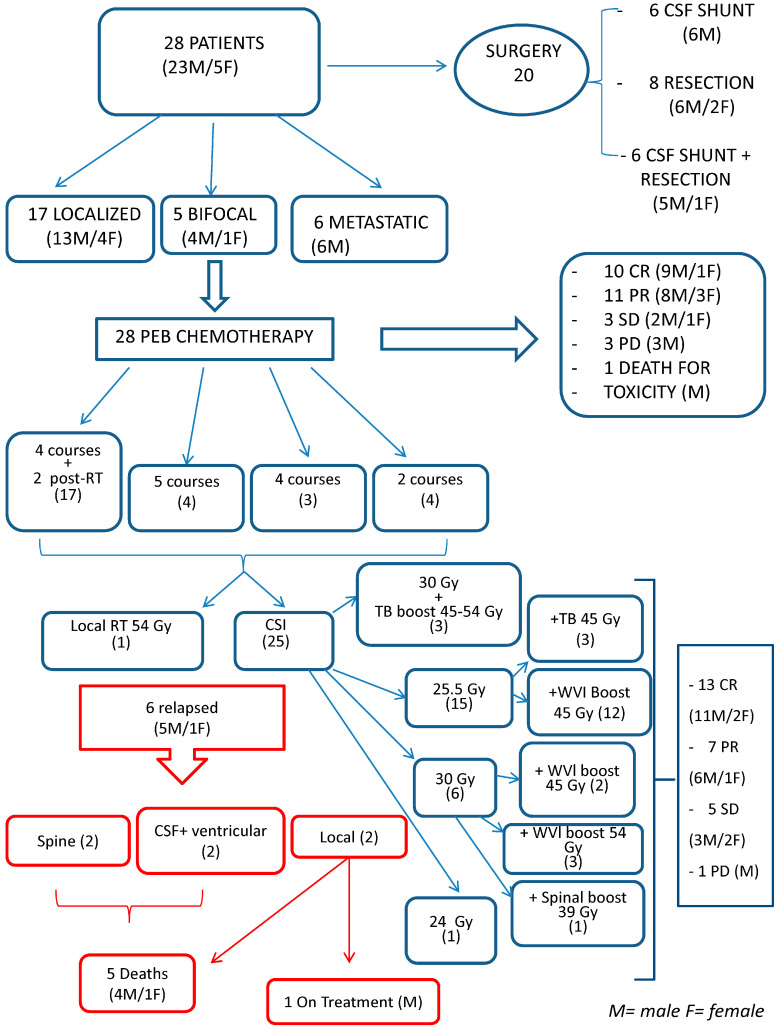
Consort diagram of the whole series.

The consort diagram in Figure 1, where patients’ sex is also clarified, summarizes the main points discussed in this section.

### 3.3. Chemotherapy

Seventeen out of 28 patients (61%) received four plus two 2 PEB cycles. Four received five cycles (due to severe hematological toxicity after the fifth cycle in two cases, and because markers progression after the first and fifth cycles, respectively, in the other two). Three patients had only four PEB cycles, and four patients only two.

Three patients presenting with high-risk disease at diagnosis (αFP > 1000 ng/mL) had intensified chemotherapy regimens consisting of two PEB cycles followed by other sequential high-dose drugs, RT, and autologous stem cell transplantation previously harvested.

Details of PEB toxicity and schedule variations are given in Table S1.

### 3.4. Radiotherapy

RT was delivered 3–4 weeks after the end of chemotherapy. Table S2 reports the RT schedules administered. Twenty-five patients (96%) received CSI. Three had CSI 30 Gy plus a focal boost to the TB up to 45–54 Gy. Fifteen patients had a total CSI dose of 25.5 Gy, plus a boost to the TB up to 45 Gy in three cases, and a WVI boost up to a total dose of 45 Gy in 12. Six patients had CSI 30 Gy, plus a WVI boost up to a total dose of 45 Gy in two cases, and 54 Gy in three; the patient with the spinal tumor received a local boost up to 39 Gy. One patient, already treated at diagnosis with 18 Gy of stereotactic radiosurgery on TB at another institution, only had CSI 24 Gy and one had 54 Gy only on the supratentorial lesions due to previous RT at diagnosis (ex juvantibus) elsewhere. Two of the 28 patients had no RT due to rapid tumor progression and clinical deterioration after a second PEB cycle in one case and death due to *P. carinii* pneumonia after the fourth PEB cycle in the other.

### 3.5. Response

Objective response to PEB was documented in 25/28 patients (Figure 1). After four PEB cycles and before RT, 10 patients were in CR on MRI, with normalized serum and CSF markers; 11 were in PR on MRI, with normalized serum and CSF markers; and 3 had SD on MRI, with markers returning to normal after two (in one patient) and four (in two patients) PEB cycles. Three patients experienced PD (documented by MRI in one case, by markers in one case and by both in the other) after one to four cycles. One patient died of pneumonia while in CR: they had negative markers and radiological PR after 2 cycles; CR of the CNS disease was confirmed at autopsy.

All four patients with a positive CSF cytology at diagnosis were in complete CSF remission after pre-RT chemotherapy.

In total, three patients had radiological PD prior to RT. One had radiological PD after the fourth cycle, despite marker normalization, and underwent surgery; a mature teratoma was diagnosed and the patient’s treatment was concluded as prescribed. One had a clinical deterioration and radiological progression after the second cycle, and died soon afterwards. One only had markers indicating progression after one PEB cycle, and was rescued with intrathecal chemotherapy, CSI and four further PEB cycles.

Of the 26 patients receiving RT, 13 had a radiological CR, 7 PR, 5 SD and 1 PD.

Responses to chemotherapy and radiotherapy were not different in males and females.

#### 3.5.1. Results in High-Risk Patients

The three patients presenting with αFP > 1000 at diagnosis—in both CSF (5862 and 1996 ng/mL) and serum (11,069 and 2614 ng/mL) in two of them, and only in serum (1155 ng/mL) in the third all achieved humoral and radiological response after two PEB cycles. Thereafter, they had an intensified treatment consisting of sequential high-dose cyclophosphamide and/or HD-etoposide, myeloablative thiotepa, and RT, as shown in Table S3.

#### 3.5.2. Survival End-Points

With a median follow-up of 133 months (interquartile range extremes 77,190, the latest patient having 26 months of follow-up), 21 patients (75%) were alive with no clinical, humoral or radiological evidence of disease progression/relapse.

Six patients have died, five due to PD/relapse, and one due to *P. Carinii* pneumonia while in CR.

**Figure 2:**
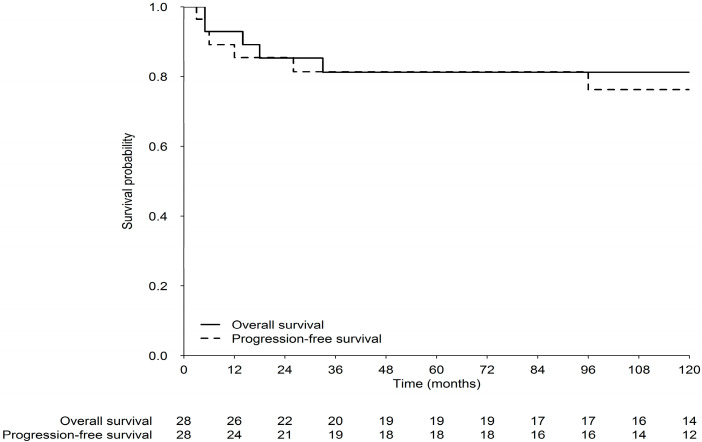
Overall survival (OS) and progression-free survival (PFS) of the whole series.

In the series as a whole, the 5 year OS (95% confidence interval [CI]) and PFS were both 81% (68–98%), while the 10 year OS and PFS were 81% and 76%, respectively (Figure 2). Again, PFS and OS were not different in males and females.

**Figure 3:**
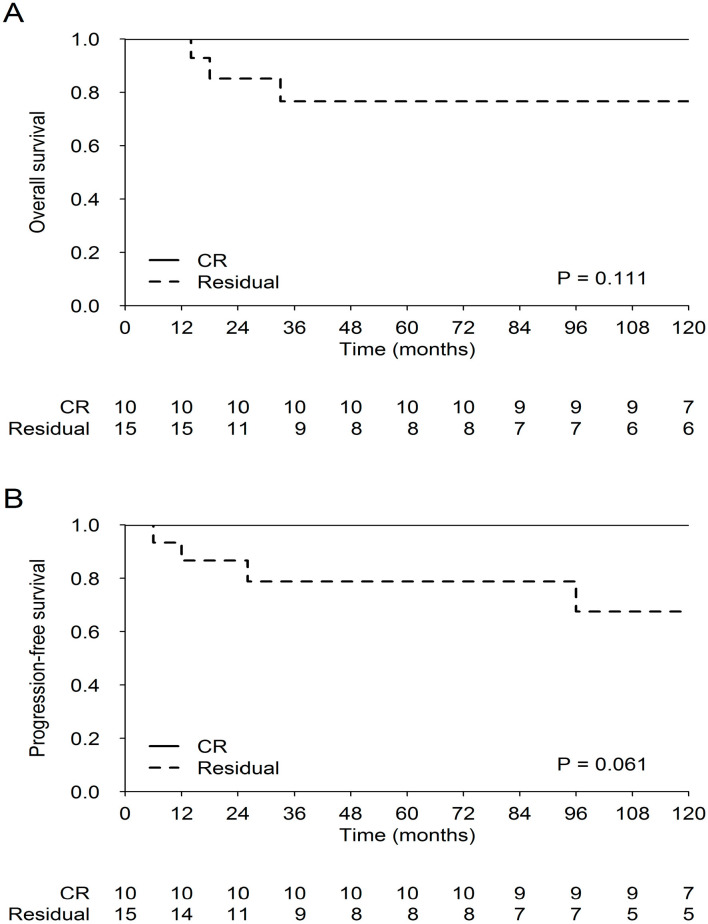
Overall Survival (**A**) and Progression-free Survival (**B**) according to response after 4 PEB (cisplatin/etoposide/bleomycin) cycles.

For the localized and metastatic disease, the 5 year OS (95% CI) was 83% (73–91%), and 82% (67–97%) [*p* = 0.13], respectively, and the 5 year PFS (95% CI) was 82% (74–90%), and 67% (48–86%) [*p* = 0.12], respectively. Comparing patients in CR after pre-RT chemotherapy with those in PR at the same time point, the survival curves confirm the excellent prognosis for the former (OS at 120 months: 100% for those in CR vs. 76% for those in PR [*p* = 0.11]; and PFS at 120 months: 100% and 67%, respectively [*p* = 0.061]) (Figure 3).

#### 3.5.3. Patterns of Relapse/Progression

Six patients experienced relapse/progression: five out of six have died and one is still in treatment after 43 months from relapse. The median time to relapse was 21 months (mean 52; range 5–162). The sites involved were: the spine in two patients (both had received CSI); local in two (before RT in one case); and CSF/ventricular seeding in two (no WVI boost had been performed in one of them).

Patients with localized disease obtaining less than CR after chemotherapy had three relapses: one local, two disseminated (one ventricles + parenchyma and one CSF only).

#### 3.5.4. Surgery and Risk Dissemination

Sixteen out of 28 patients had hydrocephalus at diagnosis, and 14/28 patients (50%) had some form of diagnostic surgery. There was a trend towards statistical significance for the association between the presence of a shunt and nodular dissemination (*p* = 0.057), and the association between the presence of a shunt and CSF dissemination at diagnosis was statistically significant (*p* = 0.024).

#### 3.5.5. Long-Term Assessment

Twenty-one patients underwent audiometry at least once during their follow-up, revealing normal hearing function in 13, and various degrees of bilateral neurosensory hypoacusia in 8 (requiring bilateral hearing aids in 1). Spirometric data were available for 18 patients, showing normal values in 12, a slight restrictive pulmonary deficit in 4, and a more severe scenario (mimicking a restrictive deficit) in 2. Twenty-one patients had thyroid gland sonography, revealing a normal thyroid in 19, and a hypoplastic parenchyma in 2. One patient developed a meningioma, one a cavernous angioma, and one a focal nodular hyperplasia of the liver.

All 22 surviving patients are being actively followed up. Four are unemployed (one developed an organic psychosis and one has a psychomotor delay with daytime somnolence after a transient ischemic attack occurring 18 years after treatment), while the others have a job or are continuing their formal education.

#### 3.5.6. Neurocognitive Evaluation

The data available for the patients in our series are reported in Table S4. Patients had a borderline mean FSIQ (83), with a significant (>10) gap between their PIQ (76.50) and VIQ (92.33). Executive functioning was at the lower end of the normal range. Visuospatial memory (−3.03) and visual-motor integration (−4.46) showed significant delays (>2 SD below the normative mean). The results of neurocognitive tests are reported in Table S5.

## 4. Discussion

### Of All GCTs, NGGCT Carry the Worst Prognosis

Most of the epidemiological data available report a male/female ratio of around 4, as the series here discussed, with no impact on survival [17,18]. A hypothetical influence of hormonal status could be hypothesized but has not so far been described.

About 10% of patients with intracranial GCTs present with insidious symptoms, and the diagnosis is delayed. Proactive diagnostics and early detection are important for these tumors because a delayed diagnosis can negatively influence patients’ survival [19]. In our series, the mean (9 months) and median (60 days) duration of symptoms before diagnosis was consistent with the diagnostic delays reported in the literature [20]. The need for emergency/diagnostic surgery should be considered with caution due to the risk of early tumor dissemination, as underscored in the recent SIOP CNS GCT II protocol [6], and in the literature [21]. In our cohort, there was a trend towards an association between the presence of a shunt and nodular dissemination, and also a statistically significant correlation between the need for shunt and CSF dissemination at diagnosis. The obvious conclusion, even considering our small sample, is that morbidity and mortality from unnecessary neurosurgery should be avoided [22], in line with recent protocols for NGGCTs [6].

After the introduction of chemotherapy in the armamentarium of GCT treatment [14,15], two later trials published in 1996 and 1998 failed to support the efficacy of chemotherapy alone, as one in two patients relapsed [23,24], but RT alone was not effective either [25]. The more recent European SIOP-CNS-GCT-96 trial enrolled 149 NGGCT patients from 11 European countries between 1996 and 2005 [26]. They received four courses of dose-intense cisplatin-based chemotherapy (cumulative doses: cisplatin 400 mg/m^2^, etoposide 1.2 g/m^2^ and ifosfamide 30 g/m^2^) followed by focal RT (54 Gy) in nonmetastatic cases, or CSI (30 Gy + 24 Gy boost) in metastatic patients. The reported 5 year OS rates were 75% and 82% for metastatic and nonmetastatic cases, respectively, and the 5 year EFS were 68% and 72%.

We here report 83% and 87% 5 year OS for metastatic and localized disease, respectively, while 5 year PFS was 67% and 93%. Our results are similar to the SIOP ones, probably meaning that we had overtreated a group of non-metastatic and non-high-risk patients since we gave CSI to all. It is noteworthy, however, that the cumulative 10 year OS and PFS rates for our series were 81% and 76%, respectively, underlining that the response was maintained over time. While CSI is judged to be unavoidable for metastatic NGGCTs, the optimal volumes for the localized disease have varied over time. Prior to the publication of the SIOP-CNS-GCT-96, a French group adopting focal field irradiation reported an EFS of 67% [27]. The North American study ACNS0122 obtained excellent results with six chemotherapy cycles (carboplatinum/etoposide and ifosfamide/etoposide) before CSI, with 5 year PFS and OS rates of 84% and 93%, respectively [8]. These authors concluded that the regimen they described should form the backbone of further studies. Brian De et al. examined instead the outcomes of patients treated with reduced-volume RT (only tumor or ventricles) at a single institution: estimates of the 4 year OS and PFS for the 14/16 evaluable patients were 92% and 81%, respectively [28]. The results of the recently published North American ACNS1123 trial better delineate patterns of failure and identified patient subgroups likely to benefit from this reduced-dose/volume RT approach [29]. After six chemotherapy cycles, patients with localized NGGCTs obtaining CR or PR after chemotherapy were treated with reduced-dose and reduced-volume RT, i.e., 30.6 Gy to the whole ventricular field with a 54 Gy TB boost (instead of the 36 Gy CSI plus 54 Gy tumor-bed boost used in the ACNS0122 trial). The 3 year PFS and OS were 87.8% and 92.4%, as compared with 92% and 94.1%, respectively, in ACNS0122 [8,29]. The authors concluded that patients with localized NGGCT achieving a CR or PR after chemotherapy and followed by reduced RT had an encouraging PFS, similar to that of patients given CSI in ACNS0122. The authors also described 10 recurrences among 66 patients given reduced-field RT, all involving the spine (but 2/10 were retrospectively judged ineligible for reduced-field RT). This pattern of failure led the COG leadership to early closure of the study accrual in September 2016. The patients without recurrent disease had nonetheless been spared full-dose CSI and, awaiting the longitudinal neurocognitive/behavioral data of the ACNS1123 study, we can assume that this could have reduced the risk of late effects and potentially improved patient quality of life. The authors concluded that the optimal RT technique, dosage and volumes for NGGCT have however yet to be ascertained [29].

In our series, among the 26 patients evaluable with MRI after RT, 1 had PD, 13 had CR, and 12 achieved PR/SD; 3 patients in the latter group subsequently developed PD and died. No second-look surgery was used for children with visible tumor after RT. There are some important issues to consider. First of all, all irradiated patients’ markers had normalized before RT. Second, the role of any residual lesion on MRI after treatment has yet to be clarified. In our experience, it remained stable over time, while in recent wider experiences (SIOP-CNS-GCT-96 and SIOPGCT II), residual disease influenced prognosis, prompting the suggestion that residual lesions following chemotherapy be resected to maximize the chance of local tumor control [26].

As documented in recent papers [8,29], and also in our series, patients with localized disease obtaining CR after chemotherapy had a better prognosis, and might be candidates for reduced RT.

The main aim of NGGCT management is still to improve OS, however. As reported elsewhere, and in agreement with the recent SIOP-GCTII protocol [6], serum or CSF αFP levels > 1000 ng/mL defined our “high-risk” group requiring treatment intensification.

The limitations of the present study include the single-institution and retrospective design, and small sample size. Other limitations are partly due to the long period of accrual, the difficulty of retrospectively reviewing imaging and assessing responses to chemo- and radiotherapy and the paucity of neurocognitive assessments (to clarify the impact of hydrocephalus, surgery and WVI). The issue of how to distinguish between CR and very good PR is still debated in the scientific community, and the adoption of radiological guidelines for this purpose has been encouraged, especially when the pineal gland is involved [30].

A series of 28 patients could be, however, considered quite a large cohort of NGGCT only (whereas some papers report on GCTs and NGGCTs together), and unlike other mono-institutional series, they had a very long follow-up, over 11 years. We found encouraging survival data for children with both localized (including bifocal) and metastatic NGGCT, after adopting an evolving on time combination of chemo- and radiotherapy.

## 5. Conclusions

NGGCTs still have a dismal prognosis. Chemotherapy and radiotherapy association is still necessary in order to ameliorate OS and EFS of these patients. Treatment intensification, including high-dose chemotherapy, is mandatory for patients presenting with high-risk disease, i.e., having alfafetoprotein levels over 1000 ng/mL at diagnosis. Defining specific subgroups of patients who could benefit from reduction of RT doses and fields is one of the main goals of future studies, together with the efforts to further improve these patients’ OS while trying to contain or avoid the long-term sequelae of chemotherapy, when feasible.

## Figures and Tables

**Table 1:** Patients’ characteristics.

Patient	Male/Female	Age at Diagnosis (yrs, Days)	L = Localized; M = Metastatic; B = Bifocal	Shunt Placement	Diagnostic Surgery
1	M	12.25	B	Y	N
2	M	13	M	Y	Y
3	M	12.17	M	Y	Y
4	M	14.5	L	N	N
5	M	13.92	L	N	Y
6	M	11.75	B	Y	N
7	M	10.33	B	N	N
8	M	12.17	L	N	N
9	M	18.42	L	N	Y
10	M	34	L	N	Y
11	M	9.75	L	Y	N
12	M	23.67	L	Y	N
13	M	23	L	N	Y
14	F	6	L	N	Y
15	M	8.75	L	N	N
16	F	10.67	B	N	N
17	M	35.67	L	N	N
18	M	8.33	M	Y	Y
19	M	15.67	B	N	Y
20	M	9.67	M	Y	Y
21	M	15.08	L	N	N
22	M	11.58	M	N	Y
23	M	15	L	Y	Y
24	M	17.33	M	Y	N
25	F	8.5	L	N	N
26	F	20.67	L	N	Y
27	M	15.75	L	Y	N
28	F	9.92	L	Y	Y

**Table 2:** Patients’ markers.

Patient	Sieric AFP Absolute Values ng/mL	Sieric AFP	CSF AFP Absolute Values ng/mL	CSF AFP	Sieric βhcg Absolute Values IU/L	Sieric βhcg	CSF βhcg Absolute Values IU/L	CSF βhcg
1	40	1	35	1	21,600	4	>10,000	4
2	0	0	0	0	14,850	4	303	2
3	11	1	119	2	0	0	0	0
4	41	1	4.6	0	680	2	171	2
5	0	0	0	0	0	0	0	0
6	432	2	207	2	0	0	8	1
7	62	1	22	1	0	0	77	1
8	0	0	0	0	8489	4	3010	4
9	394	2	145	2	450	2	372	2
10	0	0	0	0	90.98	1	147.56	2
11	0	0	0	0	148	2	280.9	2
12	11,069	4	5862	4	54	1	165	2
13	0	0	13	1	16	1	223	2
14	52	1	<1	0	1.1	0	0	0
15	0.27	0	0.44	0	10.28	1	53	1
16	1790	4	13.9	1	23	1	16	1
17	1.71	0	0.09	0	77.86	1	604	3
18	0	0	0	0	0	0	2	2
19	246	2	91	1	643	3	543	3
20	123	2	298	2	438	2	1395	4
21	0	0	0	0	46	1	257	2
22	0	0	0	0	34	1	60	1
23	221	2	not done	9	36	1	not done	9
24	1	0	46	1	49	1	500	2
25	138	2	66.5	1	360	2	414	2
26	1155	4	67.21	1	7.1	1	8.1	1
27	2614	4	1996	4	0.1	0	1.3	0
28	312.5	2	205.7	2	0.1	0	1.9	0

0 = negative, 1 ≤ 100, 2 ≤ 500, 3 ≤ 1000, 4 ≥ 1000, 9 = not done.

## Supplementary Materials

The following are available online at https://www.mdpi.com/2072-6694/12/9/2688/s1, Table S1: Details of chemotherapy and radiotherapy; Table S2: Radiotherapy details; Table S3: High-risk patients; Table S4: Neurocognitive evaluations: demographical characteristics of the sample; Table S5: Neurocognitive evaluations: mean scores of neurocognitive abilities of patients and normative means.
