# Supplementary material for: Secreting Germ Cell Tumors of the Central Nervous System: A Long-Term Follow-up Experience
Source: Cancers (Basel). 2020 Sep 21;12(9):2688. doi: 10.3390/cancers12092688 (PMC7565315; doi:10.3390/cancers12092688)
Supplement: Supplementary file 1 [file cancers-12-02688-s001.pdf]

Table S1. Details of chemotherapy and radiotherapy.

Patient	*PEB cycles*	*Deviations from the 6 scheduled PEB cycles and/or from RT schedule*
1	5	Complete surgery for PD after 4 PEB cycles, 1 PEB post-surgery, RT, PD at the 6th cycle --> intensification (Carboplatinum/Etoposide-Ifosfamide/Vincristine/HD-Cyclo, PBSC harvest, HD-VP16) --> further PD and death
2	2	PD after 2 PEB cycles and exitus
3	5	1 PEB--> markers progression--> intrathecal CT and RT --> PEB x 4
4	4	exitus for P. Carinii pneumonia after 4 PEB cycles in CR at autopsy
5	6	6th PEB cycle delivered at 75% of the dose for previous hematological toxicity
6	6	no deviations: 4 PEB--> RT --> 2 PEB
7	4	no PEB post-RT because of the pre-radiant PEB-related toxicity
8	6	no ventricular boost for stereotactic radiosurgery at diagnosis at another institution
9	5	no 2nd PEB post RT for previous toxicity
10	5	no 2nd PEB post RT for previous toxicity
11	6	no deviations: 4 PEB--> RT --> 2 PEB
12	2	2 PEB cycles --> HD-etoposide-->HD-cyclo --> RT--> HD-TT x 2
13	6	no deviations: 4 PEB--> RT --> 2 PEB
14	6	no deviations: 4 PEB--> RT --> 2 PEB
15	6	no deviations: 4 PEB--> RT --> 2 PEB
16	6	2 PEB cycles --> RT --> 4 PEB cycles
17	6	no deviations: 4 PEB--> RT --> 2 PEB
18	6	no deviations: 4 PEB--> RT --> 2 PEB
19	6	no deviations: 4 PEB--> RT --> 2 PEB
20	6	no deviations: 4 PEB--> RT --> 2 PEB
21	6	no deviations: 4 PEB--> RT --> 2 PEB
22	6	5th PEB cycle delivered at 75% for previous hematological toxicity, 6th cycle at full dose
23	6	no deviations: 4 PEB--> RT --> 2 PEB
24	6	6th PEB delivered at 75% for previous hematological toxicity
25	6	no deviations: 4 PEB--> RT --> 2 PEB
26	2	2 PEB cycles --> HD-cyclo --> HD-TT x 1 due to poor PBSC rescue --> RT
27	2	2 PEB cycles --> HD-cyclo --> HD-TT x 2 --> RT
28	4	4 PEB cycle (pre-RT only)

LEGENDA: PEB= cisplatin/etoposide/bleomycin; RT= radiotherapy; PD= progression disease; HD= high dose ; PBSC= peripheral blood stem cell ; VP16= etoposide ; CT= chemotherapy;TT= thiotepa ; cyclo= cyclophosphamide

Table S2. Radiotherapy details.

Patient	*RT fields*	*Total dose CSI/boosted site*
1	CSI + WVI	CSI 25.5 Gy/WVI 45 Gy
2	-	-
3	CSI+ T (pineal gland)	CSI 30 Gy/T 45 Gy
4	-	-
5	CSI + WVI	CSI 25.5 Gy/WVI 45 Gy (Boost 21.6)
6	CSI + WVI	CSI 25.5 Gy /WVI 45 Gy
7	CSI+WVI+TB (pineal gland)	CSI 25.5 Gy/WVI 45 Gy/Pineal gland 19.8 Gy
8	CSI	CSI 24 Gy
9	CSI + WVI	CSI 25.5 Gy/WVI 45
10	CSI + WVI	CSI 25.5 Gy/WVI 45 Gy
11	CSI + WVI	CSI 25.5 Gy/WVI 45.6 Gy
12	CSI + WVI	CSI 30 Gy/WVI 45 Gy
13	CSI + TB (lumbar lesion)	CSI 30 Gy/Lumbar lesion 39 Gy
14	CSI + WVI	CSI 25.5 Gy/ WVI 45 Gy
15	CSI + WVI + TB (hypophyseal peduncle)	CSI 25.5 Gy/ WVI+T 45 Gy

16	TB	54 Gy
17	CSI + WVI	CSI 25.5 Gy/ WVI 45 Gy
18	CSI + WVI	CSI 30 Gy /WVI 54 Gy
19	CSI + WVI	CSI 30 Gy/ WVI 54 Gy
20	CSI + WVI	CSI 30 Gy/WVI 54 Gy
21	CSI + TB (hypothalamic-hypophiseal region)	CSI 30 Gy/T 54 Gy
22	CSI + TB (hypophyseal peduncle)	CSI 30 Gy/T 45 Gy
23	CSI + WVI	CSI 25.5 Gy/WVI 45 Gy
24	CSI + WVI	CSI 30 Gy/WVI 45 Gy
25	CSI + WVI	CSI 25.5/WVI 45 Gy
26	CSI + TB (sellar region)	CSI 25.5 Gy/T 45 Gy
27	CSI+ TB (pineal gland)	CSI 25.5 Gy /T 45 Gy
28	CSI+ TB (pineal gland)	CSI 25.5 Gy /T 45 Gy

RT= radiotherapy; ***T***= tumor site

CSI= craniospinal irradiation; ***TB***= tumour bed

WVI= whole ventricular irradiation; ***Gy***= Gray

Table S3. High-risk patients.

Patient	**Age at diagnosis (years)**	**Serum AFP ng/mL**	**CSF AFP ng/mL**	**Serum βhcg IU/L**	**CSF βhcg IU/L**	**RT fields**	**Total dose (Gy)**	**PEB cycles**	**Treatment schedule**
12	23.67	11069	5862	54	165	CSI + WVI	30/45	2	2 PEB , HD-VP16, APHERESIS, HD-CYCLO--> RT--> HD-TT x 2
26	20.67	1155	67,21	7.1	8.1	CSI + TB	25.5/45	2	2 PEB , HD-CYCLO, APHERESIS, HD-TT x 1(poor PBSC harvest)--> RT
27	15.75	2614	1996	0,1	1.3	CSI + TB	25.5/45	2	2 PEB , HD-CYCLO, APHERESIS, HD-TT x 2-->RT

AFP= alpha-fetoprotein; ***HD***= high dose; ***CSF***= cerebrospinal fluid; ***VP16***= etoposide; ***Gy***= Gray ; ***CYCLO***= cyclophosphamide; ***RT***= radiotherapy; ***TT***= thiotepa; ***PEB***=cisplatin/etoposide/bleomycin; ***TB***= tumour bed **;** ***CSI***= craniospinal irradiation; **PBSC**= peripheral blood stem cell; ***WVI***= whole ventricular irradiation

Table S4. Neurocognitive evaluations**: d**emographical characteristics of the sample.

Demographical characteristics		
	n (%)	***Mean (SD)***
Gender (male)	4 (66.70%)	
Age at diagnosis [months]		132.33 (42.87)
Time since diagnosis [months]		60.17 (57.39)
Age at evaluation [months]		192.50 (48.58)

Table S5. Neurocognitive evaluations: mean scores of neurocognitive abilities of patients and normative means

	Whole sample		***Normative means***	
Neurocognitive domain	***Mean***	***(SD)***	***Mean***	***(SD)***
Intellectual abilities				
FSIQ	83.00	(15.44)	100.00	(15.00)
PIQ	76.50	(15.80)	100.00	(15.00)
VIQ	92.33	(19.42)	100.00	(15.00)
Attention				
CPT Overall Index			< 8	
Executive Functioning				
WCST Errors	89.83	(17.21)	100.00	(15.00)
WCST Perseverations	91.50	(15.99)	100.00	(15.00)

Memory				
Rey Memory	-3.03	(0.76)	0.00	(1.00)
Visual-motor integration				
Rey Copy	-4.46	(3.94)	0.00	(1.00)
